# The complete mitochondrial genome of *Pelodiscus axenaria* (Testudines: Trionychidae)

**DOI:** 10.1080/23802359.2019.1623132

**Published:** 2019-07-10

**Authors:** Xiao-Ming Yu, Yan-Feng Lin, Li-Fang Peng, Yong Zhang, Shun-Qing Lu, Song Huang

**Affiliations:** a College of Life and Environment Sciences, Huangshan University, Huangshan, China;; b Fishery Bureau of Xiuning County, Huangshan, China

**Keywords:** Mitogenome, *Pelodiscus axenaria*, phylogeny

## Abstract

The complete mitochondrial genome sequence of *Pelodiscus axenaria* was determined by shotgun sequencing. The total length of mitogenome is 16,593 bp, and contains 13 protein-coding genes, 22 tRNA genes, 2 ribosome RNA genes, and 1 control region. Most of the genes of *P*. *axenaria* were distributed on the H-strand, except for the ND6 subunit gene and eight tRNA genes which were encoded on the L-strand. The phylogenetic tree of *P*. *axenaria* and 11 other closely related species was reconstructed. The phylogenetic analyses based on these mitogenomes presented here will be useful for further insights on the evolutionary relationships of Trionychidae.

*Pelodiscus axenaria* (Zhou, Zhang and Fang, 1991) was firstly reported as a new species by Zhou Gong-jian et al. in Hunan province in 1991. In 2019, this species was found in Xiuning, Huangshan, Anhui province (Lin et al. in press). It is a small species in this genus and inhabits in the upstream rivers or mountain streams. In this research, we determined and described the mitogenome sequence of *P*. *axenaria* in order to obtain basic genetic information about this species.

The specimen of *P*. *axenaria* was collected from Xikou town, Xiuning county, Huangshan, Anhui, China, in May, 2017. It was preserved and deposited at the Museum of Huangshan University (Voucher numbers: H2). Total genomic DNA was extracted from muscle using a Qiagin DNEasy blood and tissue extraction kit (Qiagen Inc., Valencia, CA). The complete mitogenome sequence has been submitted to GenBank with the accession number MK867844.

The complete mitochondrial genome sequence of *P*. *axenaria* has been obtained from shotgun sequencing. The total length was sequenced to be 16,593 bp, which consisted of 13 typical vertebrate protein-coding genes (PCGs), 22 transfer RNA (tRNA) genes, 2 ribosomal RNA (rRNA) genes, and 1 control region (D-loop). The base composition was 35.7% for A, 28.3% for T, 11.4% for G, and 24.6% for C. The positions of RNA genes were predicted by the MITOS (Bernt et al. 2013), and the locations of protein-coding genes were identified by comparing with the homologous genes of other related species. Most of the *P*. *axenaria* mitochondrial genes are encoded on the H-strand except for the ND6 gene and eight tRNA genes, which are encoded on the L-strand. Among the mitochondrial protein-coding genes, the ATP8 was the shortest while the ND5 was the longest. The gene order, contents, and base composition are identical to those found in typical vertebrates (Boore 1999; Sorenson et al. 1999).

The phylogenetic analysis of *P*. *axenaria* was reconstructed based on the complete mtDNA sequences with other 11 related species from GenBank by MEGA 7.0 (Kumar et al. 2016) using maximum-likelihood (ML) methods. The ML tree (Figure 1) was reconstructed in http://www.phylo.org/portal2/login!input.action. The *P*. *axenaria* was close to *P*. *sinensis*. The phylogenetic analysis result was consistent with the previous research with a high support. It indicated that our new determined mitogenome sequences could meet the demands and explain some evolution issues.

**Figure 1.**
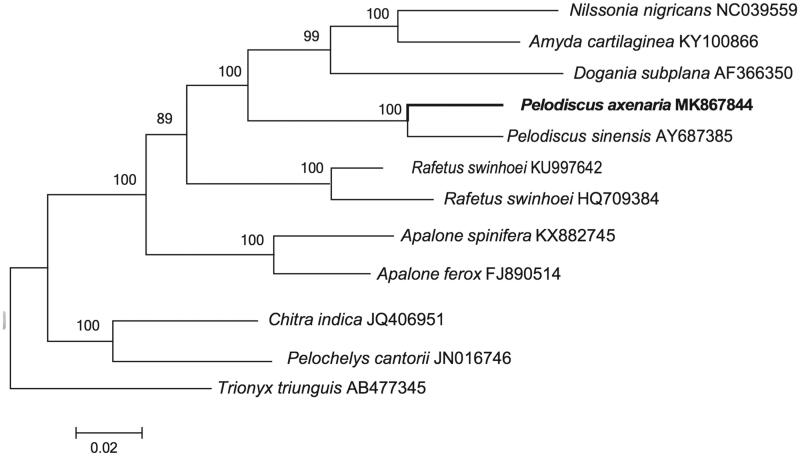
A maximum likelihood (ML) tree of *Pelodiscus axenaria* in this study and 11 related species was constructed based on the dataset of the whole mitochondrial genome by online tool RAxML. The numbers above the branch meant bootstrap value. Bold black branches highlighted the study species and corresponding phylogenetic classification.

## References

[CIT0001] Bernt M, Donath A, Juhling F, Externbrink F, Florentz C, Fritzsch G, Putz J, Middendorf M, Stadler PF 2013 MITOS: improved de novo metazoan mitochondrial genome annotation. Mol Phylogenet Evol. 69:313–319. 22982435 10.1016/j.ympev.2012.08.023

[CIT0002] Boore JL 1999 Animal mitochondrial genomes. Nucleic Acids Res. 27:1767–1780. 10101183 10.1093/nar/27.8.1767 PMC148383

[CIT0003] Lin YF, Peng LF, Gan CX, et al. in press *Pelodiscus axenaria* discovered in Xiuning County, Huangshan City, Anhui Province. Chin J Zool.

[CIT0006] Kumar S, Stecher G, Tamura K 2016 MEGA7: Molecular Evolutionary Genetics Analysis version 7.0 for bigger datasets. Molecular Biology and Evolution. 33:1870–1874. 27004904 10.1093/molbev/msw054 PMC8210823

[CIT0005] Sorenson MD, Ast JC, Dimcheff DE, Yuri T, Mindell DP 1999 Primers for a PCR-based approach to mitochondrial genome sequencing in birds and other vertebrates. Mol Phylogenet Evol. 12:105–114. 10381314 10.1006/mpev.1998.0602

[CIT0007] Zhou GJ, Zhang XJ, Fang ZG 1991 Bulletin of a new species Trionyx. Acta Sci Nat Univ Norm Hunan. 14:379–382.

